# Vaccination in children with allergy to non active vaccine components

**DOI:** 10.1186/s40169-014-0043-0

**Published:** 2015-02-14

**Authors:** Fabrizio Franceschini, Paolo Bottau, Silvia Caimmi, Giuseppe Crisafulli, Liotti Lucia, Diego Peroni, Francesca Saretta, Mario Vernich, Carlotta Povesi Dascola, Carlo Caffarelli

**Affiliations:** Pediatric Unit, “Ospedali Riuniti”, University Hospital, Ancona, Italy; Pediatric Unit, Imola Hospital, Imola, Italy; Pediatric Unit, Department of Pediatrics, University of Pavia, Pavia, Italy; Allergy Unit, Department of Pediatrics, University of Messina, Messina, Italy; Pediatric Unit, Civic Hospital, Senigallia, Italy; Clinica Pediatrica Unit, University of Ferrara, Ferrara, Italy; Pediatric Unit, Palmanova Hospital, Palmanova, Italy; Pediatric Unit, Bollate Hospital, Bollate, Italy; Clinica Pediatrica Unit, Department of Clinical and Experimental Medicine, Azienda Ospedaliera-Universitaria, University of Parma, Parma, Italy; Clinica Pediatrica Unit, Department of Clinical and Experimental Medicine, Azienda Ospedaliera-Universitaria, University of Parma, Via Gramsci 14, 43123 Parma, Italy

**Keywords:** Hypersensitivity, Vaccine, allergy, Children, Egg. cow’s milk, Gelatin

## Abstract

Childhood immunisation is one of the greatest public health successes of the last century. Vaccines contain an active component (the antigen) which induces the immune response. They may also contain additional components such as preservatives, additives, adjuvants and traces of other substances. This review provides information about risks of hypersensitivity reactions to components of vaccines. Furthermore, recommendations to avoid or reduce reactions to vaccine components have been detailed.

## Introduction

Vaccines contain an active component (the antigen) and additional components. Allergy to additional components, especially foods, is not uncommon in childhood. Egg allergy affects 2.5% of infants [1], cow’s milk allergy 2.2% [2,3]. Despite a long-lasting debate, the approach to vaccination in children allergic to additional constituents remains one of ambiguity and concern for most pediatric health care professionals. The Committee of the Italian Pediatric Society ofAllergology and Immunology selected for their expertise in research and clinical practice of management of vaccination in at risk children, convened in April 2013 to establish the structure of this document. Three panelists were delegated to draft a document addressing the identified key questions on commercially available vaccines. The document was based on agreement between panelists and on a comprehensive overview of the literature. A careful evaluation of the literature was performed. PubMed database was queried for articles published in the last 10 years describing allergic reactions following vaccination. The literature search was undertaken in May 2014. The following search terms were included: vaccine, adverse effect, adverse event, allergy, hypersensitivity, cow’s milk, egg, yeast, gelatin, dextran, thimerosal, antibiotic, adjuvant, stabilizer, latex, aluminium, phenoxyethanol. The search was limited to articles in English, humans and age 0–18 years. Articles were considered if they described systematic reviews, meta-analysis, randomized-controlled trials, observational studies, and case reports. The search found 1937 references. Articles, including those on ancient vaccines, that were useful for the purpose of this article were cited. Relevant articles published before 2004 were either familiar to the authors or they were selected by searching the reference lists of identified articles. The expert panel convened in June 2014 to review distinct aspects of the document and to reach consensus for the final document.

## Review

### Clinical approach

The vaccinator must carefully evaluate, prior to vaccine injection, if any risk of developing hypersensitivity or allergic reactions exists to prevent potentially severe hypersensitivity reactions. Therefore parents must be asked whether the child has ever experienced any allergic sign or symptom after a vaccination even if they were not reported or diagnosed to be an allergy. Vaccinator should be trained to recognize when the referred reactions were allergic. Furthermore, in the pre-vaccine anamnesis, all prior adverse reactions must be addressed, including those to additional vaccine components such as: culture media antigens, adjuvants, preservatives, stabilizers, antimicrobial agents, additives [4,5]. When an adverse reaction to a vaccine or to its constituent is reported, more specific issues have to be investigated: route of administration (oral or parenteral), clinical signs and symptoms, time and severity of reactions, treatment and therapies provided. Severe asthma and anaphylaxis must be ruled out: these conditions always require an hospital supervised vaccination [6]. In a suspect of allergic reaction to vaccine or its components arises, patients need to be referred for an allergy consultation before vaccination.

### Potential allergens

Any vaccine contains culture media antigens, which are protein-based, and could be responsible for hypersensitivity reactions (Table 1).

**Table 1:** **Reactions to vaccine components in studies with more than 25 participants**

**Vaccine component**	**Studies**	**Partecipants, N**	**Allergic reactions N (%)**
Egg	Measles-mumps-rubella		
	Goodyear-Smith et al. 2005 [9]	73	0 (0%)
	Erlewyn-LajeunesseM et al. 2008 [10]	100.000	42 (0.04%)
	Govindaraj P et al. 2009 [11]	45	0 (0%)
	Cronin J et al. 2012 [12]	310	6 (1.9%)
	Andersen DV et al. 2013 [13]	32	0 (0%)
	Total	100.460	48 (0.04%)
	Influenza		
	Cerecedo C et al. 2007 [27]	26	0 (0%)
	Esposito S et al. 2008 [28]	44	3 (6.8%)
	Chung E et al. 2010 [29]	171	29 (16.9%)
	Gagnon R et al. 2010 [24]	830	9 (1.08%)
	Greenhawt MJ et al. 2010 [30]	105	0 (0%)
	Pien GC et al. 2010 [31]	62	0 (0%)
	Webb et al. 2011 [32]	152	2 (1.3%)
	Erlewyn-Lajeunesse M et al. 2011 [21]	3640	88(2.41%)
	Howe et al. 2011 [25]	135	0 (0%)
	Owens et al. 2011 [33]	64	4 (6.25%)
	Shuler JE et al. 2011 [34]	62	4 (6.4%)
	Fung et al. 2012 [35]	56	2 (3.5%)
	Greenhawt MJ et al. 2012 [26]	105	0 (0%)
	Wainwaring Upton JE et al. 2012 [36]	77	0 (0%)
	Des Roches et al. 2012 [23]	367	13 (3.54%)
	Forsdhal BA et al. 2012 [37]	80	3 (3.75%)
	Total	5982	157 (2.62%)
Yeasts	DiMiceli L et al. 2006 [45]	107	15 (14%)
Dextran	Zanoni G et al. 2008 [62]	100.000	19 (0.01%)
Thimerosal	Wattanakrai P et al. 2007 [72]	46	0 (0%)

### Egg

Egg allergy is one of the most frequent food allergy in infants. Although egg tolerance could spontaneously develop, about 60% of chidren with egg allergy do not tolerate egg at 6 years of age [7]. Egg proteins, especially ovoalbumin, could be present in several vaccines prepared on embryonated chicken eggs. Egg proteins concentrations are not usually reported and could vary among vaccine’s brands, batches and cultivation’s protocols. Concentrations are usually higher on embryonated chicken eggs vaccines (influenza, yellow fever, rabies) and are lower on chicken embryos or fibroblasts of chicken embryos vaccines (Measles-Mumps-Rubella, tick-borne encephalitis). The European Union legislation has established 2 μg/ml as the maximum egg proteins concentration allowed, which has been demonstrated to be a safe amount in patients with previous egg’s anaphylaxis. Therefore, no significant risk of allergic reactions following vaccination should be foresighted in patients with egg’s allergy [8], even though all scientific studies referred to oral rather than parenteral contact [8].

- *MMR vaccine* - Measles-Mumps-Rosolia (MMR) vaccine is grown on cell cultures employing chicken embryo-fibroblasts. This vaccine contains minimal amounts of egg proteins, from nanograms to picograms, often not precisely reported. Therefore, this vaccine shows a very low risk of allergic reactions, even in egg’s anaphylaxis [9-13] (Table 1). In Europe, prevalence of anaphylaxis due to MMR vaccine is estimated at 1.2 cases per million doses [14]. In the US, an incidence of 3.5 cases per 1,000,000 doses has been reported [15]. Interestingly, the vast majority of allergic reactions to MMR is observed in patients without egg allergy [16]; hence, it is probable that the real triggers are other vaccine’s components, such as gelatin [17]. Available data show no additional risk for immediate reactions in egg-allergic children, compared to non allergic, with commercial MMR or measles vaccines. In the Italian National Health Instiute website it is clearly reported that all children with egg-allergy must be vaccinated and no specific precautions are needed, not even in previous egg anaphylaxis [18]. However, a review [16] that analyzed all severe allergic reactions to MMR referred from 1966 to 1999, reported 14 local reactions and 16 systemic reactions in 1803 egg-allergic children undergone to MMR vaccine. None of these reactions was fatal. In 3 out of 16 systemic reactions a proper anamnesis was collected and it was positive for a previous severe allergic reaction after egg ingestion and/or a chronic asthma. Therefore, it has been suggested, in case of severe previous egg anaphylaxis or moderate reactions in chronic active asthma, to administer the vaccine under hospital supervision. Sensitivity and specificity of skin prick test with MMR vaccine are low and not predictive of serious reactions, thus are not recommended [19,20].
- *Influenza vaccine* - Despite a theoretical risk of hypersensitivity reactions to minimal amount of egg-proteins [21,22] in influenza vaccine, studies, including papers and abstracts, from 1977 to 2012 showed that in 4172 egg-allergic patients (and 513 with severe allergic reaction), safety of influenza vaccine has been proven, with especial regard to both severe anaphylactic reactions (respiratory distress or hypotension) and mild reactions (urticaria or mild wheezing) [23]. These allergic reactions rates did not differ significantly from those in non-egg-allergic controls [24-26]. In investigations published in the last ten years [21,23-37] the frequency of allergic reactions to vaccine was 2.62% in 6532 subjects (Table 1). The American Academy of Paediatrics [19] has recently stated that the risks of missing influenza vaccination outweigh those from vaccination itself. Therefore, egg allergy of any severity (including anaphylaxis) is not a contraindication to the administration of influenza vaccine. However, influenza vaccine should be administrated when resuscitation facilities are available, and the patient should be observed in the office for 30 minutes after immunization [19]. In egg-allergic patients the injectable vaccine should be preferred rather than intranasal preparation because of an higher amounts of ovalbumin in the latter [5]. Influenza vaccine contains less than 1 μg/dose of ovalbumin. Those with an higher amount (more than 1.2 μg/mL) should be avoided in egg-allergic subjects [38]. Influenza vaccine cultured on human cells or insect culture are not approved for pediatric use.
- *Administration of influenza and MMR vaccines* - All egg-allergic children can receive influenza or MMR vaccine in primary care physician’s office. Or in vaccination centre provided with appropriate equipment and medications and with a 60 minutes post-vaccine observation (Table 2). It is recommended to investigate all suspected egg allergy before vaccination. Due to low sensitivity and specificity, skin prick test with influenza vaccine is not recommended. As well, divided doses of vaccine is not required because even in most severe egg-allergy vaccine can be tolerated in full dose. Children with a previous history of severe reactions to egg (anaphylaxis) should instead receive their vaccine in a hospital supervision [16]. This recommendation, however, shows a low grade of strength and should be reviewed in the future guidelines [39].

**Table 2:** **Vaccination protocols in allergic subjects**

**Allergen**	**Vaccine**	**Vaccination protocol**	**Setting**
Egg	Yellow fever* Rabies* ° Influenza MMR Tick-borne en- cephalitis	MMR and Influenza:	
		1) If egg-allergy normally administer with a 60 minutes observation	1) Office
		2) If egg-anaphylaxis normally administer with a 60 minutes observation	2) Hospital
		Yellow fever:	
		1) If skin tests are negative: normally administer with a 60 minutes observation	Hospital
		2) If skin tests are positive: desensitization/graded doses	
Cow’s milk	OPV, DTP, DT, DTaP, PCV-13	If previous anaphylaxis normally administer with a 60 minutes observation	Office
Yeasts	Hepatitis B, Quadrivalent HPV, meningococcal , PCV-13, typhoid (oral)	1) If skin tests with vaccine are negative: normally ad- minister with a 60 minutes observation	Hospital
		2) If skin tests with vaccine are positive: desensitiza- tion/graded doses	
Neomycin	MMR, IPV, rabies, influenza, varicella, Zoster HepA	1) If local skin reaction: normally administer	Office/Hospital
		2) If anaphylactic reaction: no vaccine	
Gelatin	MMR*, Varicella*, Zooster*, Yellow fever* Rabies °, DTP Influenza	1) If skin tests are negative: normally administered with a 60 minutes observation	Hospital
		2) If skin tests are positive: gelatin-free vaccine or de- sensitization/graded doses	
Latex	When vaccine has no removable contaminated part (prefilled syringe), vaccine should be normally administered with a 60 minutes observation		Hospital

*High amount. °To be used when polygeline free and egg free vaccine is unavailable.
- *Yellow fever vaccine* - Yellow fever is an acute viral disease caused by Flavivirus and transmitted by the bite of mosquito (*Aedes Aegypti*). It is endemic in South America and Africa and has an high mortality. Yellow fever vaccination is, therefore, required for traveling in endemic countries. Yellow fever vaccine is grown in chicken embryos and contains higher amounts of egg-proteins in comparison with MMR and influenza vaccines. Unlike influenza and MMR vaccines, all subjects with egg-allergy and previous systemic reactions should receive yellow fever vaccine under hospital supervision. Patients with egg allergy should be evaluated before vaccination, with skin prick test and serum specific IgE to eggs [39]. When there is an history of anaphylaxis to egg, it may be performed a skin prick test with a 1/10 dilution of vaccine, and, if negative, with undiluted vaccine. If skin prick tests are negative, an intradermal test should be performed with a 1/100 dilution (1/10 dilution has been demonstrated to be irritative) [39]. If all skin tests are nega-tive, vaccine could be normally administered, with a 60 minutes post-vaccine observation (Table 2). If skin tests are positive, vaccine should be injected in graded dose under hospital supervision. It has been demonstrated that an intradermal 1/5 dose of vaccine could be protective and safe in egg allergy subjects who have shown a severe local urticaria reactions [40].
- *Rabies vaccine* - A rabies vaccine without egg and gelatin is available. Therefore such vaccine should be administered to subjects with severe allergy to egg protein or gelatin (Table 2).

### Milk

Milk proteins are often used as stabilizers or emulsifiers in food derivates. Some vaccines (Table 2) could contains hidden milk proteins, in order to prevent viruses degradation. Anaphylactic reactions have been reported in milk and egg-allergic children after MMR vaccination [41]. Kattan JD et al. [42] have evaluated 8 pediatric patients with previous anaphylaxis arisen within 60 minutes after an acellular diphtheria-tetanus-pertussis vaccine. In 6 of these children an immediate allergic reaction to milk proteins have been recorded, and in 5 of these the reaction was a severe one. In all children a significant sensitization to milk proteins have been documented within 2 years after vaccine reaction. Some researchers have observed that culture media used for commercial vaccine against *Chlostridium tetani*, *Corynebacterium diphteriae* and *Bordetella pertussis* could have been supplemented with aminoacids derived from the hydrolysis of milk proteins. Authors have demon-strated, with an ELISA essay, a concentration between 8.1 and 18.3 ng/mL of casein peptides in 8 different batches of DTP (Diphteria-tetanus-pertussis) vaccine [43]. Furthermore, hidden amounts of alpha-lactalbumin have been detected even in some oral polio vaccine (OPV) [44]. Four children, receiving OPV and measles-mumps vaccine at the same time, have shown severe systemic reactions after vaccines injections. In these children there was a previous history of milk proteins allergy but no egg-allergy. As well, skin prick test and serum specific IgE were positive for milk proteins but negative for egg proteins. Moreover, skin prick test with OPV, but not for measles-mumps vaccine, were positive.Although based on these scattered case-studies, due to the possible presence of milk proteins in OPV and DPT vaccine, it has been suggested, in children with history of milk’s proteins anaphylaxis, a 60 minutes observation after vaccination (Table 2) [43].

### Yeasts

Yeast proteins could be present as *Saccharomyces cerevisiae* derived antigens in hepatitis B vaccine (up to 25 mg/dose) and in quadrivalent human papillomavirus vaccine (less than 7 mcg/dose) [39]. Yeast is also contained in pneumococcal 13-valent conjugate (PCV-13), in meningococcal and in oral thyphoid vaccines. Allergic reactions to yeasts proteins appear to be rare. In the US, in more than 180,000 vaccine adverse reactions, only 15 were resulted from a reaction to yeast proteins. Furthermore, these 15 cases may have been even related to other vaccine components [45]. Although rare, in children with proven yeasts allergy, it is recommended to prefer yeast-free Vaccine (e.g. bivalent human papillomavirus vaccine). If not available, these children should be evaluated with skin prick tests and serum specific IgE dosage to *Saccharomyces cerevisiae* before vaccination. If an IgE sensitization is confirmed, children could receive graded vaccine; if negative, vaccine could be administered in the usual manner, with a 60 minutes observation after injection (Table 2).

### Additives

#### Stabilizers

Stabilizers are used to protect vaccine from excessive heat, drying, antigens’ adherence to vial’s walls. More common stabilizers are sugars (destran, lactose, saccharose), aminoacids (glycin, monosodium salt of glutamic acid) and proteins (gelatin, human serum albumin).

### Gelatin

Gelatin is a partial hydrolyzed protein derived from animal connective tissue. It is an ubiquitous antigen: it is commonly used in foods, juices and wines, confectionery and it is also used for pharmaceutical purposes in tablets, caps, suppository, plasma expanders, collagen, stitches. Gelatin for medical purpose is of bovine or porcine origin and shows an extensive cross-reactivity (although not with fish derived gelatin) [46]. It is used as a stabilizer in several vaccines that are currently available with concentrations between 15 μg and more than 15,500 μg/dose of vaccine. [20,47]. The highest concentrations of gelatin are contained in MMR, rabies, varicella-zoster, oral typhoid and yellow-fever vaccines, and, in less amounts (up to 2,000 μg/dose) in DTP and influenza vaccines. Gelatin is the vaccine component responsible for most allergic reactions to vaccine, for both IgE and non IgE mediated reactions [48]. Severe reactions due to gelatin have been described for MMR [47,49], varicella [50-52], yellow fever [49] and japanese encephalitis vaccines. European and US studies have documented gelatin specific IgE in 14-28% of patients with vaccine anaphylaxis, while Japanese studies reported much higher data (86-100%) in patients with MMR and varicella vaccines anaphylaxis [47,53]. These differences could be explained by the different type of gelatin used in Japan in 1990. In those years, in the US MMR vaccine contained highly hydrolyzed porcine gelatin with low molecular weight. In Japan gelatin was of bovine origin, partially hydrolyzed and with a small amount of high molecular weight; highly hydrolyzed porcine gelatin was used from 1998 and was completely removed from DTP vaccine since 1999. It is possible, however, that genetic issues should be taken into account. It has been demonstrated that subjects with HLA-DR9 haplotype carry a 4 times increase risk to develop gelatin specific IgE [54].

However this does not mean that they have clinical hypersensitivity reactions to gelatin. Nowadays vaccines contain highly hydrolyzed porcine gelatin, which shows a lower sensitization rate with a subsequent decrease of vaccines allergic reactions [55]. For the above mentioned reasons, every child who needs a gelatin-containing vaccine should be investigated for previous or possible gelatin-allergic reactions, especially with food ingestion. Children sensitized to red-meat (bovine, pork, lamb) show a higher risk: 16% of children sensitized to bovine meat and 38% of children sensitized to pork meat already have specific IgE to gelatin. This sensitization increases the risk of vaccine allergic reactions [56]. One study has highlighted that immediate allergic reactions to bovine meat usually arise in the first year of life and involve 6% of children affected by atopic dermatitis or food allergy and 20% of children with milk proteins allergy [57]. In some subjects even delayed allergic reactions could be due to red meat (3 to 6 hours after ingestion). These subjects have a positivity to serum specific IgE for bovine, pork and lamb meat and milk, and mild positivity to skin prick test with commercial extract (wheal less than 4 mm). On the contrary, they have a strong positivity to skin prick test with fresh meat [58]. In these patients serum IgE for galactose-alpha-1,3-galactose are detectable. This latter compound is a carbohydrate found in mammals (not in primates or humans) and it is considered, by some authors, as the major gelatin allergen [56]. All children with a previous immediate reaction to food gelatin should undergo an allergy consultation. However, patients without gelatin allergy may show allergic reactions to gelatin-containing vaccine [49]. This could be explained by the different route of exposure to gelatin allergens. Serum specific IgE are available for gelatin, while no commercial standardized skin prick extract to gelatin is available. Skin prick test could be performed with a prick-by-prick technique dissolving 1 spoon of sugared gelatin powder in 5 mL of saline solution (non sugared powder tends to became a gel at room temperature) [6]. If the skin prick test is negative but a suggestive history of gelatin-allergy is reported, an oral provocation test should be suggested [59]. If the oral provocation test is positive, a skin prick test with the gelatin-containing vaccine should be performed and, if negative, the vaccine could be normally given with a 60 minutes observation. If the skin prick test is positive, a gelatin-free vaccine should be used or, if not available, vaccine should be injected in graded doses (Table 2) [5] in a setting with resuscitation equipment [60]. For example, for a 0.5 mL vaccine, the following doses may be given at 15–30 minutes intervals [20]:0.05 mL 1:10 dilution;0.05 mL full-strength;0.1 mL full-strength;0.15 mL full-strength;0.2 mL full-strength.

### Dextran

Dextran has been associated to anaphylaxis following both Bacillum Calmette Guerin, due to a IgG mediated reaction with complement fixation [61], and a MMR vaccine that is no longer available [62]. Delayed localized skin reactions have been reported, such as maculopapular exanthemas, urticarial vasculitis and neutrophilic dermatosis [63].

### Preservatives

Preservatives are usually added to vaccines preparation to prevent bacterial or fungal growth or contamination.

- *Thimerosal -* Thimerosal is an organic mercurial (46% mercury) compound, metabolized to ethyl-mercury and thiosalicilate. This preservative has been used by pharmaceutical corporations since 1930 for ophthalmological, dermatological, otolaryngological purposes, for endovenous immunoglobulin, and vaccine preparation. Previously available vaccines containing thimerosal were DTP, *Haemophilus Influentiae*, DT, hepatitis B, Influenza, *Neisseria Meningitis*, *Steptococcus Pneumoniae* and Rabies vaccines. In 1998 the “Centers of Disease Control and Prevention” has recommended the complete removal of thimerosal from vaccine, because of a possible cerebral toxicity induced by this preservative. Indeed, the cumulative amount of methyl-mercury to which children were exposed in the first 6 months of life exceeded the recommended dose of 187,5 mcg, that has been assessed as inoffensive [64]. From 2001, in developed countries, thimerosal has been removed from pediatric vaccines. Nevertheless, some preparations of Td, DT, influenza (mainly in multi dose vials), meningococcal (multidose vials) vaccines could contains minimal, not significant, trace amounts (<1 mcg/0,5 ml). However mercury’s toxicity has never been fully demonstrated by scientific studies, and toxic levels were extrapolated from studies conducted on methyl-mercury’s toxicity, which is way more toxic and has a half-life 7 times less than mercury [65]. Current evidence sustains the safety of the use of thimerosal as a preservative for inactivated vaccines [66] . This supports the administration of multidose vials of thimerosal-preserved vaccines, especially in low- and middle-income countries, where they are a critical part of immunization programs [67]. Thimerosal’s sensitization could be investigated by patch tests and it is detectable in 10% of the general population. It is more frequent in those countries that are more exposed to thimerosal containing preparations, in patients affected by atopic dermatitis or conjunctivitis and in subjects treated with desensitization thimerosal-containing therapies [68]. Thimerosal is responsible for delayed hypersensitivity reactions, among which atopic dermatitis and local skin injection reactions [69]. Only one generalized cellular-mediated reaction has been reported after influenza vaccination [70]. Even in patch positive subjects, exposure to thimerosal is considered safe [68,71] and no contraindications are reported [72].
- *Phenoxyethanol –* 2-phenoxyethanol (2-PE) is an antibacterial additive associated with delayed hypersensitivity reactions. Sensitization’s rate to 2-PE is pretty low, local allergic reactions are rare and no systemic reaction has been reported [73].
- *Antibiotics***-** Many vaccines contain little amounts of antibiotics such as neomycin, aminoglycosides, streptomycin, polymixin, tetracycline, and the fungicide amphothericin B, to prevent bacterial and fungal contamination. No vaccine contains beta-lactams or sulfonamides. Until now, only a few case-reports of vaccine adverse reactions due to antibiotics has been reported [5]. Although, a proven anaphylaxis or allergy to one or more antibiotics contained in vaccines is considered an absolute contraindication to those vaccines.

### Adjuvants

Aluminium salts (aluminium hydroxide, aluminium phosphate, aluminium potassium sulphate) are widely used as adjuvants in vaccines and in allergy immunoterapy, due to their immunogenic effect. This activity, once thought to be due to “depot” effect, is probable caused by an increased activity of antigen presenting cells or by an increase of cytokines and complement production [74]. DTP, hepatitis A and B, *Haemophilus Influentiae B* vaccines are prepared adsorbing the antigens in either an aluminium hydroxide or aluminium phosphate gel. As an alternative, antigens are precipitated in a solution of alum. Interestingly, children are anyhow exposed to aluminium salts since they are also detectable in breast milk and in artificial powder milk. No anaphylaxis to aluminium salts have been reported. The most known and frequent reaction is a palpable nodule at the injection site [75]. Sterile abscesses [76], localized or systemic dermatitis due to aluminium salts have been reported [53].

## Contaminants

### Latex

Latex could be detected in vaccine’s vial or syringe. A contamination of vaccines by latex particles could rarely induce hypersensitivity reactions in latex-allergy children, this risk seems to be mild. In a 160,000 case-review of vaccine adverse events in the US, only 28 cases could have been attributed to an IgE mediated latex-allergy [77]. Nowadays, most vaccinal preparations are latex free. If a known latex-allergic patient requires a vaccine only available with latex contamination, it is suggested to remove the cap from the vial and to avoid the contact between the cap and the needle. If a contaminating piece could not be removed or avoided, normally administer the vaccine and have the patient under a 60 minutes supervision (Table 2) [41].

## Conclusions

Children with allergy to non-active components of vaccines are rare. When they receive a vaccine containing the constituent to which they are hypersensitive, the risk of having an allergic reaction is small. However, this risk should not be neglected since a high number of doses are administered. Vaccinators should be able to identify children at risk for allergic reactions to vaccine. An allergological evaluation may be sought in selected cases to assess how and when vaccines may be given. In subjects at high risk, the vaccine can be safely given with precaution, when necessary giving administering doses, and with available materials and support for the treatment of anaphylaxis. This may avoid unnecessary incomplete vaccinations.
